# Pesticide Residues in Organic and Conventional Agricultural Soils across Europe: Measured and Predicted Concentrations

**DOI:** 10.1021/acs.est.3c09059

**Published:** 2024-04-03

**Authors:** Dennis Knuth, Lingtong Gai, Vera Silva, Paula Harkes, Jakub Hofman, Marek Šudoma, Zuzana Bílková, Abdallah Alaoui, Daniele Mandrioli, Igor Pasković, Marija Polić Pasković, Isabelle Baldi, Mathilde Bureau, Francisco Alcon, Josefa Contreras, Matjaž Glavan, Nelson Abrantes, Isabel Campos, Trine Norgaard, Esperanza Huerta Lwanga, Paul T. J. Scheepers, Coen J. Ritsema, Violette Geissen

**Affiliations:** † Soil Physics and Land Management Group, Wageningen University and Research, Droevendaalsesteeg 3, 6708 PB Wageningen, The Netherlands; ‡ RECETOX, Faculty of Science, Masaryk University, Kamenice 753/5, 625 00 Brno, Czech Republic; § Institute of Geography, University of Bern, Hallerstrasse 12, 3012 Bern, Switzerland; ∥ Cesare Maltoni Cancer Research Center, Ramazzini Institute, Via Saliceto 3, 40010 Bologna, Bentivoglio, Italy; ⊥ Department of Agriculture and Nutrition, Institute of Agriculture and Tourism, K. Huguesa 8, 52440 Poreč, Croatia; # Univ. Bordeaux, INSERM, BPH, U1219, 146 Rue Léo Saignat, 33076 Bordeaux, France; ∇ Escuela Técnica Superior de Ingeniería Agronómica, Universidad Politécnica de Cartagena, Paseo Alfonso XIII, 48, 30203 Cartagena, Spain; ○ Agronomy Department, Biotechnical Faculty, University of Ljubljana, Jamnikarjeva 101, 1000 Ljubljana, Slovenia; ◆ CESAM and Department of Biology, University of Aveiro, 3810-193 Aveiro, Portugal; ¶ CESAM and Department of Environment and Planning, University of Aveiro, 3810-193 Aveiro, Portugal; †† Department of Agroecology, Aarhus University, Blichers Allé 20, 8830 Tjele, Denmark; ‡‡ Radboud Institute for Biological and Environmental Sciences, Radboud University, Heyendaalseweg 135, 6525 AJ Nijmegen, The Netherlands

**Keywords:** soils, pesticides, PECs/MECs, farming systems

## Abstract

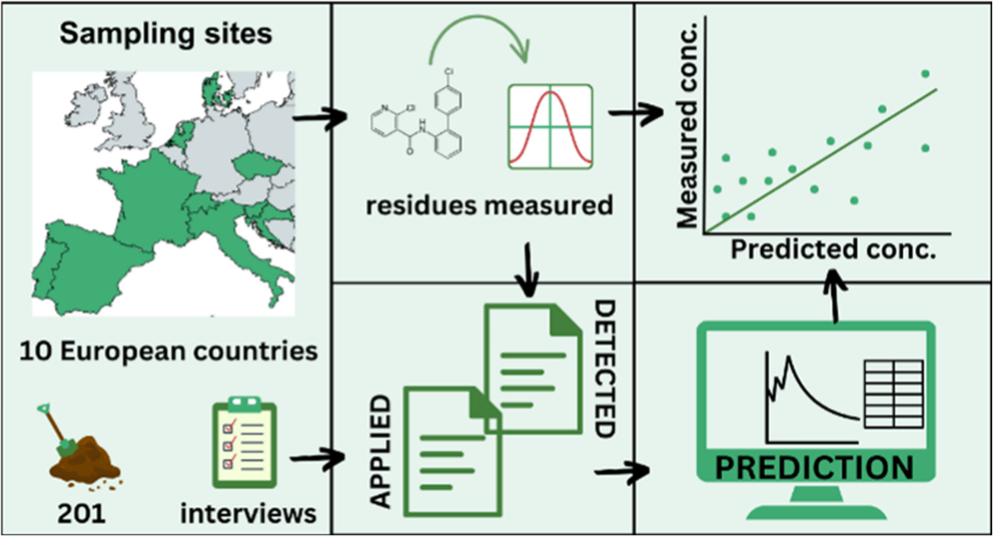

During the growing season of 2021, 201 soil samples from conventionally and organically managed fields from 10 European countries and 8 cropping systems were taken, and 192 residues of synthetic pesticides were analyzed. Pesticide residues were found in 97% of the samples, and 88% of the samples contained mixtures of at least 2 substances. A maximum of 21 substances were found in conventionally managed fields, and a maximum of 12 were found in organically managed fields. The number and concentration of pesticide residues varied significantly between conventional and organic fields in 70 and 50% of the case study sites, respectively. Application records were available for a selected number of fields (*n* = 82), and these records were compared to the detected substances. Residues from 52% of the applied pesticides were detected in the soils. Only 21% of the pesticide residues detected in the soil samples were applied during the 2021 growing season. From the application data, predicted environmental concentrations of residues in soil were calculated and compared to the measured concentrations. These estimates turned out not to be accurate. The results of this study show that most European agricultural soils contain mixtures of pesticide residues and that current calculation methods may not reliably estimate their presence.

## Abstract 2

This article describes the plant protection product contamination of European agricultural soils and compares the findings to the application records of the analyzed samples.

## 1 Introduction

In recent decades, agricultural production has increased dramatically, bolstered by the use of plant protection products (PPPs). The global market value of PPPs increased from USD 20 billion in the early 1990s to USD 40 billion in the late 2000s.^1,2^ Since the 1990s, 325 000–400 000 tons of PPPs have been applied annually in the European Union alone.^3^

This intensive use of pesticides has come under scrutiny, with concerns about residue accumulation and adverse effects on nontarget organisms,^4−8^ even at recommended PPP application doses.^9^ In Europe, the risk of the PPP active substances has been assessed predominantly based on individual active substances (at the member state level, tank mixes are considered). Most assessments, including the European risk assessment for PPP market approval,^10^ do not consider the combined impact of the active substances from single pesticides with PPP residues already present in agricultural soils. Therefore, they do not take the real-life effects of environmental mixtures (“cocktail effect”) into account.^11^ Several monitoring studies found that mixtures in soil contain not only residues from currently used PPPs^12,13^ but also residues from PPPs that have been banned, some for over a decade.^14−16^ Additive or synergistic effects of such environmental mixtures on soil life have scarcely been studied.^6,17^ The co-occurrence of PPP residues, and the insufficient risk assessment of these mixtures, leaves a knowledge gap that is yet to be filled.^11^

One major limitation in almost all of the soil monitoring studies is the lack of PPP application records,^12−16^ leading to uncertainty in the prediction or interpretation of measured levels of PPP residues in soils. The residues in soil from current applications can be modeled using the predicted environmental concentration (PEC). PECs are based on persistency, displayed by the degradation time (DT_50_) and the degradation kinetics of a substance. The DT_50_ values of substances can vary vastly between different assessments.^18^ This variation in DT_50_ and the resulting uncertainty can lead to an over- or underestimation of the concentration of PPP residues in soil and therefore to a flawed risk assessment since persistence plays an important part in assessments.^19^

In this study, we investigated the occurrence of the residues of synthetic PPPs (192 substances, 152 active substances, 39 transformation products, and 1 synergist) in 201 agricultural soils across Europe. The samples originated from fields under conventional or organic management from 10 European countries, explored under the Horizon 2020 funded SPRINT project. For the selected fields, we calculated the PECs for the residues of the applied PPPs for the 2021 growing season and compared them to the measured environmental concentrations (MECs) of these PPP residues. The aim of the study was to assess PPP residues in soils across the main European cropping systems and compare the residues detected in the field with the PECs calculated by using the approach used for the approval of pesticides.

As the prevention and reduction of soil pollution is a relevant part of several current EU policies,^20,21^ this research can provide valuable insights for the Common Agricultural Policy and the proposed European Commission’s soil health law. Moreover, the results can shed light on the appropriate length of the transition period from conventional to organic pest management. These insights are particularly relevant to the Farm-to-Fork strategy, which aims to get at least 25% of European agricultural land under organic management by 2030.^22^

## 2 Materials and Methods

### 2.1 Case Study Sites and Sampling Campaign

The sampling campaign was performed in 10 European countries: Croatia (HR), Czech Republic (CZ), Denmark (DK), France (FR), Italy (IT), The Netherlands (NL), Portugal (PT), Slovenia (SL), Spain (ES), and Switzerland (CH), which all function as case study sites (CSSs). In total, 201 soil samples were taken during the 2021 growing season from 8 cropping systems across Europe (Table 1). In each CSS, organic and conventional fields were investigated. The number of fields varied between 17 and 24 per CSS, including at least 7 organic and 8 conventional fields. Further information is provided in Silva et al. and Silva et al.^23,24^ The sampling campaign was harmonized so that the majority of PPP treatments were conducted at the time of sampling in each CSS. One composite sample was collected from each field. The composite samples were created from 5 subsamples, collected from randomly selected positions in the field. Soil samples were taken from the upper 5 cm of soil in fields with permanent crops and the upper 20 cm of soil from arable land with tillage. Subsequently, subsamples were mixed and stored at −20 °C until further use. All samples were frozen, stored, and transported to the laboratory for final analysis. Before the PPP residue analysis, the samples were thawed and homogenized. CSS characteristics and sampling details are summarized in Table 1. Organic fields were under organic management for at least 5 years prior to the sampling campaign.

**Table 1:** Specification of the Case Study Sites (CSSs) of the SPRINT 2021 Sampling Campaign^a^

country	code	organic management	conventional management	crop	sampling time	sampling depth (cm)
Switzerland	CH	10	10	Fruits^b^	June–July	0–5
Czech Republic	CZ	13	11	Oilseeds^c^	May–July	0–20
Denmark	DK	10	10	Cereals^d^	May–June	0–20
Spain	ES	10	10	Broccoli	October–November	0–20
France	FR	10	7	Grapes	June–July	0–5
Croatia	HR	10	10	Olives	September	0–5
Italy	IT	10	10	Vegetables^e^	October–November	0–20
Netherlands	NL	10	10	Potatoes	June	0–20
Portugal	PT	8	12	Grapes	July–August	0–5
Slovenia	SL	10	10	Maize	September	0–20

a The number of differently managed fields, crop, sampling time, and depth.

b Apples, pears, cherries, and strawberries.

c Rapeseed, sunflower seed, mustard seed, and poppy seed.

d Spring barley, winter wheat, winter rye, winter barley, and oats.

e Radicchio, black cabbage, peppers, green cabbage, salad, and broccoli.

The main characteristics of the sampled soils (pH, bulk density, and soil organic matter) and the methods used to determine these measurements are described in the Supporting Information (Table S1). These were assessed by CSS teams following harmonized protocols.

### 2.2 Selected PPP Residues

In this study, the residues of synthetic PPPs were analyzed. They were selected based on prescreening conducted by the SPRINT project. This selection of substances contained 50 herbicides, 57 fungicides, 45 insecticides, 39 metabolites, and the synergist piperonyl butoxide. Of these substances, 111 (57.8%) were approved (01.01.2021) for use in the EU, 42 (21.8%) were not approved, and 37 (19.3%) were metabolites and napropamide (M) with a pending approval state. Represented crops are displayed in Table 1. The criteria for the selection of analytes and crops have been published.^23^

The PPP residues were extracted using the KOH extraction method (glyphosate and AMPA) previously described by Bento et al. and Yang et al., and the other PPP residues were extracted with a modified QuEChERS method.^25,26^ Both methods are described in detail in the Supporting Information (Supporting Information I, Supporting Information II, Table S2 and Table S5).

### 2.3 Data Analysis

The limit of detection (LOD) was set as the reporting value. All values above the LOD with quality concerns were discarded.

Statistics were performed with R. After checking for normal distribution using the Kolmogorov–Smirnov test and using Levene’s test to check the homogeneity of variances, the Kruskal–Wallis test was performed to compare the number of substances detected and their total concentration between organic and conventional fields within each country and between countries. For pairwise comparisons between the farming systems within a CSS, Mann–Whitney U tests were performed.

Application records of the 2021 growing season, for 82 fields, 76 conventional and 6 organic, were used to compare which PPPs were applied and which PPP residues were detected. This comparison was performed at field level leading to 15 744 possible detections. The number 82 was obtained after a thorough quality control performed by Mark et al. (in prep),^27^ which included a comparison of farmers’ application records and the technical leaflets of the products mentioned to be applied. The application records include records from all 10 countries.

### 2.4 Calculation of PECs

The PECs at the time of sampling were calculated using the process-based Single First Order, Double First Order in Parallel, and First-Order Multi-Compartment degradation kinetics implemented in the mkin R package version 1.1.1.^28^ The PECs were calculated for each field using available application records provided by the farmers (Mark et al.).^27^ Following the EFSA PEC calculation approach used for the approval of pesticides in the EU, a generic value of 1.5 g cm^–3^ was used for the bulk density.^29^ A table linking the plant growth stages to interception was used to determine the specific crop interception at the date of the PPP application (Table S6). For permanent crops, the interception was assumed to be 70% for olive trees^29^ at every point in time, 65% for fruit before May 15th and 80% after, and 40% for grapes before April, 50% until May 10th, and 70% afterward. The resulting interception values were 97% (*n* = 77) in line with the available interception values provided by Mark et al. (in prep.)^27^ for the applications on trees and grapes. If available, degradation kinetics and parameters were derived from the respective EFSA report for each substance (Table S7); for DT_50_, the worst-case field study value was used. When no degradation kinetics were available, Single-First-Order decay was used as a default. For 38 applications on fields originating from the same farm, it was not clear which application was applied on which field. In these cases, PECs were calculated for both fields and both results are displayed graphically but only accounted for once in all downstream analyses.

## 3 Results and Discussion

### 3.1 PPP Residues in Soil

**Figure 1:**
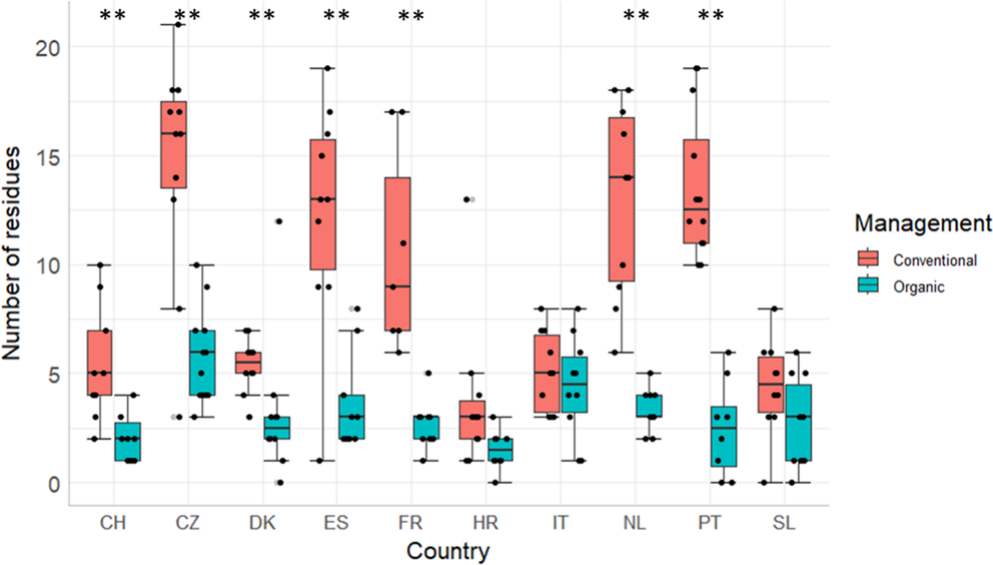
Number of PPP residues found in different farming systems (conventional = red, organic = petrol) by country (Switzerland = CH, Czech Republic = CZ, Denmark = DK, Spain = ES, France = FR, Croatia = HR, Italy = IT, The Netherlands = NL, Portugal = PT, Slovenia = SL). Black dots show the individual sample results, and gray dots denote outliers. Boxplots show median Q1 and Q3, and a 1.5 * interquartile range as min and max. If the numbers of substances detected differed between organic and conventional soils, the country is indicated by the significant levels of *p* > 0.05 = * or *p* > 0.01 = **.

In 195 of the 201 analyzed soil samples, PPP residues (at least one substance out of 192 analyzed) were detected (Figure 1). At least one substance was detected in 99% of conventional fields and 95% of organic fields. This changed to 95% in conventional and 64.4% in organic fields when residues of obsolete organochlorine substances were taken out of the analysis. Furthermore, the numbers went down to 92% in conventional and 53.5% in organic fields when residues of all banned substances were taken out of the analysis.

In further analysis, the whole data set is analyzed. PPP residue counts per sample reached a maximum of 21. The highest median number of residues across all CSSs was detected in the conventional fields from the CZ with 17, the highest number of substances detected was in conventional fields from the CZ (21), PT (19), and ES (19). The lowest median number of substances in conventional fields was detected in samples from HR with 3. The highest median number (6) of substances in an organic field was found in CZ, while the highest number (12) of substances was detected in DK. The lowest median number of residues in organic fields was observed in samples from HR and CH with 3 and 4, respectively. This is in line with Pelosi et al.^13^ (31 analytes, 180 samples from the agricultural landscape (with and without PPP application)) who found residues of at least one PPP in 100% of arable soil samples monitored. This is even higher than findings from Geissen et al.^15^ (151 analytes, and 340 soils analyzed) who found PPP residues in 98% of soils from conventionally managed fields, with up to 16 different PPP residues, and in 71% of organic European agricultural soils, with up to 6 different PPP residues. Especially for organic fields in the present study, the observation was higher, with 95% of samples having at least one detected residue. In total, 87.6% of the samples analyzed in this study contained mixtures of at least two substances. This frequency was significantly higher in conventional samples (96%) than in organic samples (79.2%). In addition, our results uncovered higher numbers than Silva et al.^14^ (76 analytes, 317 soils analyzed) who found residues present in 83% of the analyzed soil samples, and mixtures of residues in 53% of the samples. The significant difference between the number of PPP residues detected between conventional soils and organic soils in the current study is in line with the literature.^15,16^ The number of PPP residues differed significantly between the soils from conventional and organic management systems in the CH, CZ, DK, ES, FR, NL, and PT CSSs; all of which showed a higher number of different PPP residues in the soil samples collected from fields applying conventional management. The three CSSs where no significant difference was observed between organic and conventional managed fields were HR, IT, and SL; all three CSSs where the median number of residues found in conventionally managed fields was already low (≤5).

### 3.2 Frequency of Detected PPP Residues

In this study, 192 substances (154 parent compounds and 37 transformation products) were investigated. Of these, 100 (52%) substances were detected above their respective LODs (Table S8). The most frequently detected substances were p,p′dichlorodiphenyldichloroethylene (DDE p,p′), AMPA (aminomethylphosphonic acid, a degradation product of glyphosate), hexachlorobenzene (HCB), chlorpyrifos, and glyphosate with detection rates of 85.1, 39.5, 38.6, 33, and 24.2%, respectively. Except for glyphosate, these substances are known for their very high persistence in the environment and for their risk of particle-bound transport in the environment.^18^ AMPA is the only of the above-mentioned substances whose parent compound is still approved for use in the EU. The frequent detection of AMPA is in line with the findings of Silva et al.,^30^ who reported detecting AMPA in 42% of EU topsoil samples and glyphosate in 21%.

The substances with the five highest detection frequencies per CSS are displayed in Table S9. For example, in the CZ, DDE p,p’, and HCB were detected in 100% of the fields. Dichlorodiphenyltrichloroethane (DDT p,p’) was detected in 96%, and AMPA and DDT o,p’ were detected in 67% of CZ fields. In the NL CSS, HCB was present in all fields, DDE p,p’ was detected in 95%, DDT p,p’ was found in 55% of the fields, and fluopicolide was detected in 40% of the fields. DDE p,p’ was found in 100% of the fields in 3 out of 10 CSSs, and in 8 out of 10, it was the most frequently found substance. In PT, 7 substances were detected in 50% of fields, and in CZ, it was 6. The occurrence of metabolites of DDT is a common phenomenon, as DDT as well as its main metabolite DDE p,p’ are very persistent in the environment with a DT_50_ of over 1000 days.^18^ Geissen et al.^15^ detected two different DDT metabolites each in 20–50% of their analyzed samples; additionally, Silva et al.^14^ were able to not only detect but also quantify DDE p,p’ in 23% of their analyzed samples. As most of the recent European studies focus on residues of currently used PPPs,^12,13,16,31^ further subsumption of the pollution with DDT and its metabolites as well as HCB is difficult.

The analyzed substances included 111 approved active substances, 42 not approved active substances, and 37 metabolites. Depending on the study location, between 30.3 and 73% of the substances detected in conventional fields were approved. The highest percentages of approved substances were found in FR (73.0%) and PT (68.7%) (Table S10). Meanwhile, the lowest percentage of approved substances was detected in fields of CH (30.3%) and CZ (39.6%). Residues from substances not approved for use ranged from 16.2 to 64.3% in samples from fields where conventional management was adopted, with the lowest percentages 16.2 and 17.2% found in FR and PT, respectively. The highest percentages of nonapproved substances were found in CH (64.3%) and CZ (50.6%). In the samples from fields applying organic management, 0–45% of the detected residue substances were approved under conventional management. The highest percentages were found in PT (45%) and FR (25.9%), while the lowest number of residues from approved substances was detected in fields from HR and the NL, with 0 and 6.1%, respectively.

Residues from substances that were not approved ranged from 40.0% up to 90.9% in fields under organic management, with the lowest percentages, 40.0 and 48.1%, in PT and FR, respectively. The highest percentages were found in the NL (90.9%) and CH (80%) CSSs. The high percentage (45.0%) of approved PPP residues in the organic fields from PT is interesting to note because none of these compounds was a candidate for substitution (CfS). Also, of note here are metalaxyl (M), dimethomorph, and pyriofenone, which were found in over 25% of the organic fields. Additionally, despite their high LOD, glyphosate and AMPA were among the most frequently detected substances in organic fields in 7 CSS, with AMPA present in all 7 and glyphosate in 1.

All of the findings above indicate that the transition period of 2 years for farms to go from conventional to organic might not be long enough to ensure that there are no residues from substances that are categorized as low to moderately persistent. The highly persistent compounds, which are from former applications, are also problematic since they are ubiquitous. An adjusted transition period would challenge the objective of the Farm-to-Fork strategy of the EU which aims to get at least 25% of agricultural land under organic management before the year 2030.

### 3.3 Mixtures of PPP Residues

A total of 151 different mixtures of 2–21 substances were detected (Figure S1). Mixtures were detected in 96% of conventional and 79.2% of organic fields. These numbers decreased to 89 and 37.6% when obsolete OCPs were taken out of the data set for conventional and organic fields, respectively. Furthermore, they decreased to 83 and 26% when residues of all nonapproved substances were taken out of the data set for conventional and organic fields, respectively. This shows that a large part of mixtures found in organic fields are due to compounds that are not in use anymore, especially in the case of the OCPs. When parent substances and metabolites are grouped together, 137 different mixtures of 2–18 substances were found in the soil samples. DDTs were part of 91% of these mixtures, followed by glyphosate and AMPA at 48% and HCB and chlorpyriphos at 42% each. This is in line with the results of Silva et al., who detected 166 different mixtures, with AMPA and glyphosate among the five most frequent residues.^14,30^ In organic fields, a total of 47 different mixtures were detected, with DDTs (92%), HCB (45%), glyphosate, and AMPA (38%) contributing the most.

### 3.4 Residues of Approved PPPs

In this study, “approved substances” refers to active substances that were approved in the EU as of 01.01.2021, the year of the sampling campaign. Of the 21 groups (active substances and metabolites grouped together), which contribute to more than 10% of all mixtures, 17 (81%) were residues of approved active substances. These substances included 6 (35%) CfS. In the organic fields, 44% (4 out of 9) of the substances contributing to more than 10% of mixtures were residues of approved substances. Of the most common residues in organic fields from each CSS, 14 (25%) were residue-approved active substances and 10 (18%) were metabolites, while 33 (57%) were residues of PPPs that were not approved (Table S9). In organic fields, 44% of the substances detected in over 10% of mixtures were residues of approved substances and just one of those was a CfS. Showing that after 2 years of transition and years of organic management, there are still residues of approved substances that persist in soil. The origin of those residues could be from previous applications, drift from conventional fields in the vicinity or atmospheric deposition from distal sources.

### 3.5 Residues of Applied and Not Applied PPPs

**Figure 2:**
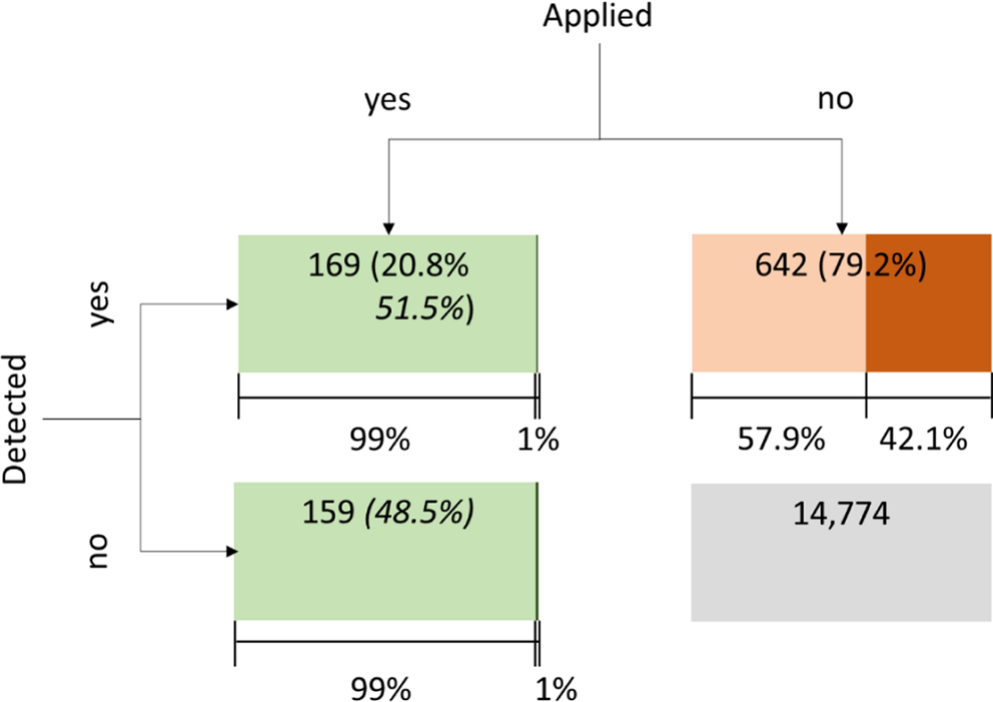
Qualitative analysis of cases of active substances applied (italic font) and substances (normal font) detected. Representative share of combined substances applied and substance detected. Light colors (green and brown) denote approved and dark colors (green and brown) denote nonapproved active substances. The gray box displays the number of cases of substances analyzed but not applied and not detected.

To compare which residues were from applied PPPs and which were detected, the application data for growing season 2021 (including applications in 2020 for winter crops) were compared to the detected PPP residues. Application data was available for 82 fields, 6 organic, and 76 conventional, which led to 15 744 (82 fields, 192 investigated substances) possible cases of PPP (residue) applications and detections. In Figure 2, the accumulated number of substances that were (a) applied and detected, (b) applied but not detected, (c) not applied but detected, and (d) not applied and not detected are displayed. Of the applied active substances, 51.5% were also detected, while 48.5% were applied but not detected. Seventy-nine percent of the substances detected were not applied. This is double the 38% found by Chiaia-Hernandez et al. in a similar analysis.^32^ It is important to note that not all of the applied PPPs were analyzed. Many detected substances were not from applied PPPs, which indicates either deposition from nearby applications or residues from previous applications in the field. The latter case could be an explanation for the discrepancy in the above-mentioned study, as the authors had application records from the farmers covering a longer time period.^32^ This is also in line with a Swiss study that found PPP residues from products that had not been used in the past decade in nearly all analyzed sites.^33^ The most common substances that were not applied but detected and approved were glyphosate, boscalid, azoxystrobin, tebuconazole, metalaxyl (M) fluopicolide, and metrafenone, with 28, 19, 16, 14, 13, 13, and 13 detections in the 82 fields, respectively (Table S11). Besides tebuconazole and fluopicolide, none of these active substances are CfS, and apart from boscalid, fluopicolide, and azoxystrobin, all are classified as non- or moderately persistent. The relatively low presence of CfS in the most detected as well as to mixtures contributing substances indicates that even the discontinuation of their use will not reduce the occurrence of PPP residue mixtures in soil substantially. The applications of nonapproved PPPs were at the CSS in HR, CH, and CZ. While the applications include two applications of thiophanate-methyl (CH and CZ) which had a maximum grace period until 19.10.2021, and thiacloprid (CH) until 03.02.2021, imidacloprid (applied in HR) had no period of grace.^34^ The high amount of detected nonapplied and nonapproved substances is highly influenced by legacy compounds, as previously described.

### 3.6 PPP Residue Concentrations

**Figure 3:**
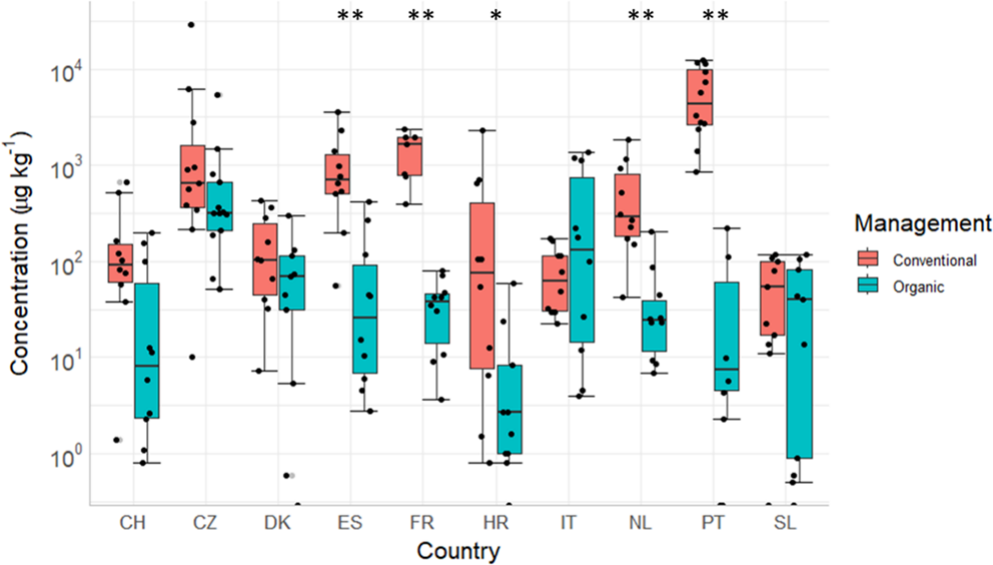
Total PPP residue concentrations in different farming systems (conventional = red, organic = petrol) divided by country (Switzerland = CH, Cech Republic = CZ, Denmark = DK, Spain = ES, France = FR, Croatia = HR, Italy = IT, The Netherlands = NL, Portugal = PT, Slovenia = SL). Boxplots show median Q1 and Q3 and a 1.5 * interquartile range as min and max. Black dots show the individual sample results, and gray dots denote outliers. With significant levels, *p* > 0.05 = * and *p* > 0.01 = **.

The total PPP residue concentrations ranged from 0.5 to 28 673 μg kg.^–1^ The highest median concentration was detected in conventional fields from PT. The highest total concentration was found in a field from the CZ. The highest median concentration in organic fields was found in the CZ (317.9 μg kg^–1^); also the highest concentration was observed in a field from CZ with 5458 μg kg^–1^. The lowest median concentrations in conventional and organic fields were observed in HR CSS (Figure 3). Significant differences in total concentrations between the farming systems were observed in ES, FR, HR, NL, and PT. In all of these CSSs, the samples from fields applying conventional management showed significantly higher total concentrations. IT was the only CSS where the organic fields had a higher total median concentration than the fields from fields using conventional management. This can be explained by high concentrations of dieldrin (in three fields over 1000 μg kg^–1^) and DDE p,p’ in some of the IT fields (Table S15).

In Figure S2a, the PPP residue levels of the soils per crop are displayed. Fruits and maize were the two crops where all soil fields showed concentrations of less than 500 μg kg^–1^. Grapes were the crop with the highest percentage of soil samples with concentrations >1000 μg kg^–1^ and showing concentrations over 10 000 μg kg^–1^. According to the application data provided by Mark et al. (in prep),^27^ fruits had, on average, the highest application rates of synthetic PPPs of all crops, followed by grapes, potatoes, and vegetables. This is not fully reflected in the measured concentrations as soils from fruit and potato fields showed relatively low concentrations. The discrepancy between the PPPs applied on fruits and the amount measured can be explained by the nature of the crops since trees grow farther from the ground and have relatively high crop interceptions.

Regarding the different climatic regions, the highest PPP residue concentrations were found in samples from southern Europe (*n* = 77) (more than 25% over 1000 μg kg^–1^) followed by northern Europe (*n* = 20) and then central Europe (*n* = 104), where 0% and less than 10% of samples exceeded 1000 μg kg^–1^ respectively (Figure S2b). Northern Europe and central Europe were also the zones where more than 50% of the soil samples had total concentrations of less than 100 μg kg^–1^. This is not in line with the results from the LUCAS project, where higher PPP residue concentrations were found in soil samples from central and northern Europe as compared to southern Europe.^35^

### 3.7 Measured and Predicted Environmental Concentrations

**Figure 4:**
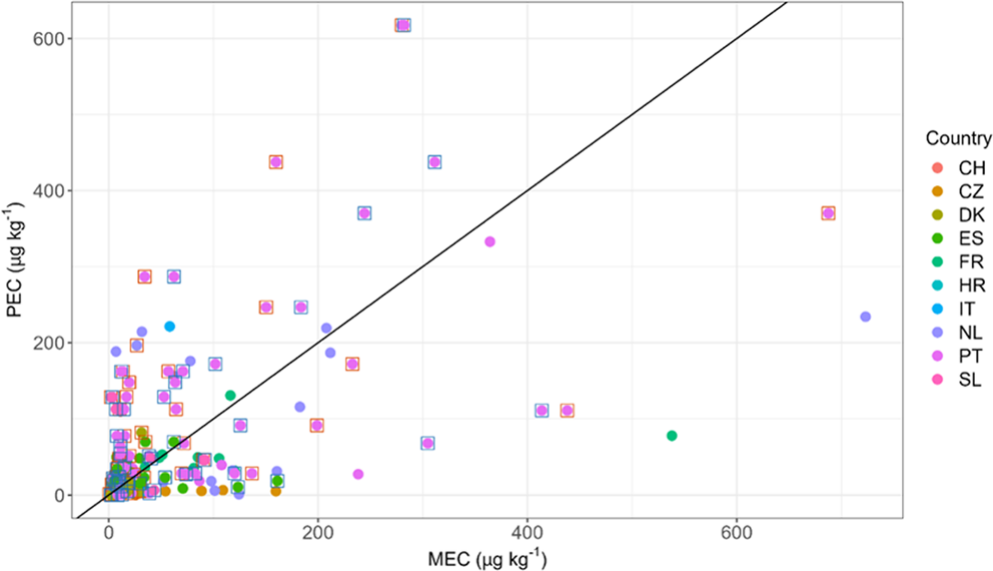
MECs vs PECs calculated based on application rates in the different CSSs (*n* = 170). A line with slope = 1 indicates PEC = MEC. Squares around the symbols indicate the origin of different field data.

In this study, we calculated the PEC for soil and compared it to the measured environmental concentration (MEC). For the calculation of PEC, only application records were used where application rate, application date, and sampling date were available (429 applications on 63 fields). The number of fields was 19 less than for the applied/detected calculations since no planting date was provided for some fields. After multiple applications of the same PPP were merged in a field, a total of 248 PECs were calculated. PECs below the LOD were excluded when there was no MEC, as the PEC would have been unmeasurably low with our methods, excluding 41 values. Of the resulting 207 PECs, 132 (64%) had corresponding MECs above the LOD. In 54 cases (41% of MEC/PEC cases, 26.1% in relation to all calculated PECs), the MEC was higher than the PEC. In 15.9%, the MEC was between 2 and 5 times higher than the PEC, in 7.5% it was between 5 and 10 times higher, and in 4.5% it was more than 10 times higher than the PEC (Figure 4). The 116 PECs with no corresponding MEC were below the LOD of the substance in 35% of the cases. The most frequently applied but not detected active substances with a PEC > LOD were deltamethrin (*n* = 9), acetamiprid (*n* = 8), metazachlor (*n* = 4), and glyphosate (*n* = 4) (Table S12). In eight of our MEC/PEC cases, the ratio was between the 0.95 and 1.05 interval (4 below and 4 above 1) representing 4% of the calculated values.

**Figure 5:**
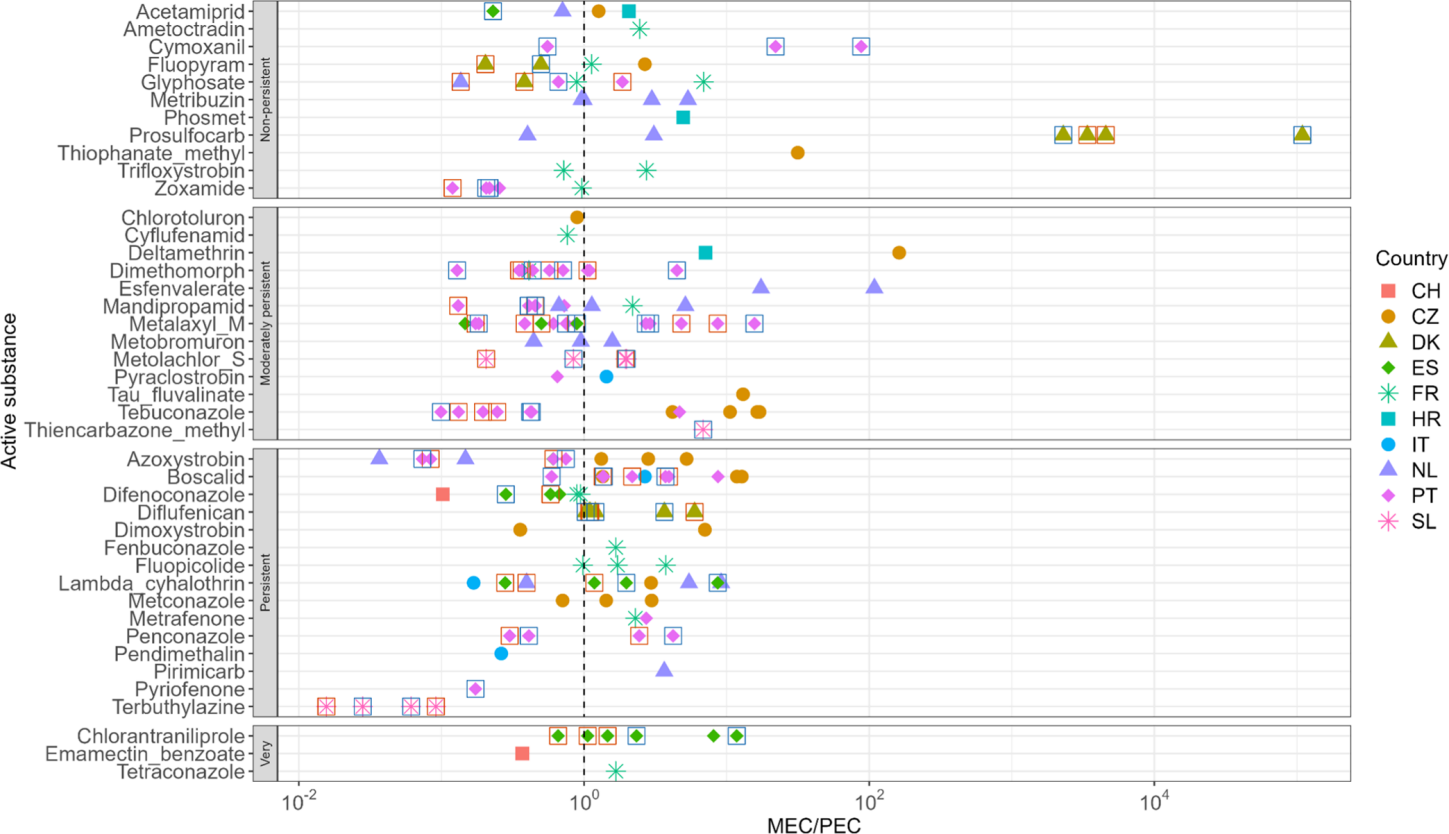
MEC/PEC ratio (*n* = 170) of different AS in the different CSSs grouped by persistency (nonpersistent = DT_50_ < 30 days, moderately persistent = DT_50_ 30–100 days, persistent = DT_50_ 101–365 days, very persistent = DT_50_ > 365 days), different symbols and colors indicate different countries, squares around the symbols indicate origin from different field data, and the dashed line indicates a ratio of 1.

As there was no sampling carried out before the growing season, it is not clear whether residues were present in the soil before collection and whether these additional residues impacted the MEC. There is no clear indication if the persistency class affects prediction precision (Figure 5). Similarly, Riedo et al. found non- or moderately persistent substances a long time after their last recorded application.^33^ Interestingly, from the residues of the four approved substances found in more than 10% of the organic samples, only boscalid had a MEC/PEC ratio >1 in more than 50% of the cases. Based on this, it could be expected that the occurrences of the other two residues found in the organic fields were a result of drift from nearby fields or distal atmospheric deposition.

### 3.8 Main Findings

In this study, we investigated the occurrence of PPP residues in 201 organic and conventional fields across 10 European countries. We compared the applied substances with the detected substances qualitatively and quantitatively. The main findings were as follows:

- Mixtures of up to 21 PPP residues were found in 96% conventional samples and mixtures of up to 12 PPP residues were observed in 79.2% of organic samples.
- In the organic samples, 40% of the residues with the highest detection frequencies per CSS were approved substances. In conventional samples, this went up to 57%.
- Total PPP residue concentrations in samples were up to 28.7 mg kg^–1^ for conventional samples and 5.46 mg kg^–1^ for organic samples.
- 79% of the detected substances were not applied to the soils sampled during the current growing season.
- For 48.5% of the PPPs applied during the growing season, no residues were detected in the samples.
- Comparison indicates that the predicted environmental concentrations, as calculated for the EU dossiers for active substance approval, were imprecise estimates of the measured environmental concentrations, and 26.1% of the calculated PECs underestimated the MECs. Only, four percent of the calculated values was in the five percent confidence interval.

These findings show that the current evaluation of PPPs is not as precise as it should be and that the longevity of non- and moderately persistent substances, which are more likely to still be approved, is underestimated. This can lead to higher total PPP residue concentrations in soil and more complex mixtures of approved and nonapproved substances, even in organic fields. For further monitoring, it is important to take application data on a field basis into account in order to see the real persistence of PPP residues in agricultural soils.

### 3.9 Limitations of This Study

Each monitoring study provides results highly dependent on the study design. The main limitations of this study can be seen in the following issues:

#### 3.9.1 Study Design

In the context of the SPRINT project, it was not possible to assess a higher number of countries, crops, and samples. Comparison between and within CSS would be easier with a more uniform choice of crops. Nevertheless, the choice of crops and countries is a not complete but good representation of agriculture in Europe, which was more desired than perfect coherency in crops.

#### 3.9.2 Varying LOD

Relatively high LODs for fluoroxypyr, glyphosate, and AMPA can lead to an underestimation of the frequency of these compounds relative to others in this study. Different LODs and LOQs also lead to difficulties in comparing studies to each other. This shows the need for harmonization in soil PPP monitoring.

#### 3.9.3 Plant Interception Estimates in PEC Calculations

For PEC calculations, estimated BBCH (Biologische Bundesanstalt, Bundessortenamt and Chemische Industrie) values and therefore interceptions had to be used, as the BBCH values were not part of the application records for each application. This can lead to inaccuracies in the estimation of the PECs, both over- and underestimations. The interceptions used were 80% in line with the interceptions provided by Mark et al. (in prep)^27^ for the cases where interception values were provided. Interception was underestimated in 8% of the estimates and overestimated in 12%. Further studies should consider assessing BBCH values for each application date in their questionnaires.

#### 3.9.4 Unknown History of PPP Application

No data were available concerning the history of PPP applications, before the 2021 growing season, for the sampled fields.

## Supporting Information Available

Soil bulk density method; pH and SOC determination; pesticide extraction method; method validation LC and GC; histogram of the number of detected PPP residues per sample by farming type (Figure S1); total PPP residue concentration per crop and region (Figure S2); references (PDF)
Soil abiotic data (Table S1); LC apparatus (Table S2); GC apparatus (Table S3); LC method (Table S4); GC method (Table S5); interception estimation (Table S6); degradation parameters for PEC calculation (Table S7); concentrations per CSS (Table S8); detection frequencies (Table S9); detection by approval status (Table S10); detected but not applied (Table S11); PECs of not detected active substances (Table S12); LC validation/recovery rates (Table S13); GC validation/recovery rates (Table S14); data set (Table S15); references (XLSX)
