# Supplementary material for: Pesticide Residues in Organic and Conventional Agricultural Soils across Europe: Measured and Predicted Concentrations
Source: Environ Sci Technol. 2024 Apr 3;58(15):6744–52. doi: 10.1021/acs.est.3c09059 (PMC11025110; doi:10.1021/acs.est.3c09059)
Supplement: Supplementary file 1 — es3c09059_si_001.pdf [file es3c09059_si_001.pdf]

Supporting information I:

Pesticide residues in organic and conventional agricultural soils across Europe: Measured and predicted concentrations

Dennis Knuth[1], Lingtong Gai[1], Vera Silva[1], Paula Harkes[1], Jakub Hofman[2*], Marek Šudoma[2], Zuzana Bílková[2], Abdallah Alaoui[3], Daniele Mandrioli[4], Igor Pasković[5], Marija Polić Pasković[5], Isabelle Baldi[6], Mathilde Bureau[6], Francisco Alcon[7], Josefa Contreras[7], Matjaž Glavan[8], Nelson Abrantes[9], Isabel Campos[10], Trine Norgaard[11], Esperanza Huerta Lwanga[1], Paul T.J. Scheepers[12], Coen J. Ritsema[1], Violette Geissen[1]

[1] Soil Physics and Land Management Group, Wageningen University and Research, Droevendaalsesteeg 3, 6708 PB Wageningen, the Netherlands

[2] RECETOX, Faculty of Science, Masaryk University, Kamenice 753/5, 625 00 Brno, Czech Republic

[3] Institute of Geography, University of Bern, Hallerstrasse 12, 3012 Bern, Switzerland

[4] Cesare Maltoni Cancer Research Center, Ramazzini Institute, Via Saliceto 3, 40010 Bentivoglio BO, Italy

[5] Department of Agriculture and Nutrition, Institute of Agriculture and Tourism, K. Huguesa 8, 52440 Poreč, Croatia

[6] Univ. Bordeaux, INSERM, BPH, U1219, 146 Rue Léo Saignat, 33076 Bordeaux, France

[7] Escuela Técnica Superior de Ingeniería Agronómica. Universidad Politécnica de Cartagena, Paseo Alfonso XIII, 48, 30203 Cartagena, Spain

[8] Agronomy Department, Biotechnical Faculty, University of Ljubljana, Jamnikarjeva 101, 1000 Ljubljana, Slovenia

[9] CESAM and Department of Biology, University of Aveiro, 3810-193 Aveiro, Portugal

[10] CESAM and Department of Environment and Planning, University of Aveiro, 3810-193 Aveiro, Portugal

[11] Dept. of Agroecology, Aarhus University, Blichers Allé 20, 8830 Tjele, Denmark

[12] Radboud Institute for Biological and Environmental Sciences, Radboud University, Heyendaalseweg 135, 6525 AJ Nijmegen, The Netherlands

[*]Corresponding author E-mail: jakub.hofman@recetox.muni.cz

Summary: This supporting information consists of 8 pages, the supporting figures Figure S1 and Figure S2.

Content:

1. Soil bulk density S3

2. PH and SOC determination S4

3. Pesticide extraction S5

4. Method validation LC and GC S6

5. Figure S1 Histogram of the number of detected PPP residues per sample S7

6. Figure S2 total concentrations of PPP residues by crop and region S7

7. References S8

1. *Soil Bulk density:*

The bulk density of the soils was assessed according to the core method.[1] For the analysis, 3 – 5 samples were taken per field. The organic layer was carefully removed to expose the mineral soil. Subsequently, a metal ring (volume between 100 - 400 cm^3) with a sharp side was put in the mineral soil layer with the sharpened side face down. The ring was manually pushed into the soil as far as possible without twisting it. Afterward, the ring was carefully hammered further into the soil to prevent compaction until the top of the ring was flush with the soil. The ring was then gently removed by pushing a putty knife under it. Finally, the ring's surface was cleaned and excess soil was cut off with a sharp knife. The samples were stored in a chamber with a relative humidity of 100%. Bulk density was determined by dividing the dry soil weight by the soil volume. For samples where no bulk density was measured (n = 17, of the French CSS) with the ring method, the data was extracted from the LUCAS data set with a resolution of 500 x 500 m.[2]

2. *pH and SOC determination:*

pH was measured using pH electrodes using a standard protocol. 10 g of air-dried fine soil (<2 mm) weighted in a centrifuge tube and 25 mL of 1 M KCl have been added. Tubes have been shaken for the extraction for 2 hours in a rotor shaker. Afterward, samples were shaken by hand twice and the pH electrode was immersed when stable the pH value was noted.[3]

For the SOC content, clean and dry porcelain crucibles were weighted. Samples were weighted in and all weights were recorded to four decimals. Approximately 1 g of finely ground rock powder was added. The weight of the crucible plus sample was noted and samples were placed in a muffle furnace for 60 minutes at 950° C. Samples were removed from the furnace and placed in a desiccator to cool down to room temperature, and weighed afterward. Percentual weight loss was calculated.

To determine the true loss on ignition for the sample: The weight percent FeO (determined by titration was multiplied by 0.111348. This value was added to the loss on ignition (whether + or -). This sum is the true loss on ignition.

3. *Pesticide extraction:*

Glyphosate and its metabolite aminomethylphosphonic acid (AMPA) were extracted similar to the procedure described by Yang et al.[4] and Bento et al.[5] Two grams of soil were extracted over 1 h with 10 mL of 0.6 M KOH in a 50 mL Grenier tube by using a vertical shaker. Afterwards, samples were centrifuged at 3500 rpm for 15 min. Subsequently, 0.5 mL of supernatant was collected in a new vial. The pH was adjusted with 40 µL of HCl (6 M) and isotope-labelled internal standard (10 µL, 10 µg L^{-1} AMPA and 10 µg L^{-1} Glyphosate) was added. For the following derivatisation 250 µL borate buffer (5%) and 250 µL FMOC-Cl were added. After shaking on a vortex, tubes were incubated at room temperature for 30 min. Then, 25 µL of formic acid was added to stop the derivatisation and samples were shaken. Finally, 0.5 mL of extract was transferred to a plastic LC vial with a 0.45 µm PTFE filter lit.

A modified QuEChERS method has been used to analyse the other pesticide residues. Five grams of soil were spiked with 10 µL of internal standard (D6-metolachlor, 10 µg mL^{-1}). Afterwards, 10 mL of Millipore water and 10 mL of acetonitrile were added, subsequently, the sample was shaken for 15 min. A salt mixture was added, containing of 4 g magnesium sulphate, 1 g sodium chloride, 1 g sodium citrate and 0.5 g disodium citrate. Subsequently, tubes were vortexed and centrifuged for 5 min at 3500 rpm. The supernatant was split to be analysed with LC-MS/MS in positive, and negative electrospray ionisation (ESI) mode, and for GC-HRMS analysis. For the LC-MS/MS method, 100 µL of formic acid (0.1%, in Millipore water) and 100 µL extract were transferred to a plastic LC vial with 0.45 µm PTFE filter lit. For the GC-HRMS analysis, a second clean-up was performed, 1 mL of extract was transferred into a 2 mL Eppendorf vial, containing a salt mixture of 25 mg PSA (primary secondary amine), 3.5 mg of GCB (graphitized carbon black and 150 mg magnesium sulphate. Eppendorf was vortexed and subsequently centrifuged for 15 min at 13,000 rpm. 100 µL of supernatant and 10 µL of injection standard (1 µg mL^{-1}, 13C-PCB-162) were transferred to a new conical vial.

4. *Method validation LC and GC:*

The chemical determinations and the quality control of the analytical results were performed according to the Document SANTE/ 12682/2019.[6]

Quality assurance and quality control samples, including blanks, standard solution and model soil samples (in house reference material with known level of pesticides) were repeatedly extracted and included in the analysis. Blanks to control for background contamination were processed in the same way as samples. All standard solutions used for the calibration curves were injected before and after each analysis of a set of samples to check for instrumental sensitivity and reproducibility. Three sets of multi-pesticide calibration standards were prepared for LC-MS/MS-based multi-method, GC-HRMS-based multi-method and glyphosate/AMPA analysis, respectively. Each set of calibration standards was prepared from a mix solution that were going to be analysed by the respective analytical method. The calibration standards for LC-MS/MS analysis were prepared in solvent (multi-method: ACN + Millipore water; glyphosate/AMPA:Millipore water) while the calibration standards for GC-HRMS analysis were matrix-matched. The calibration ranges were as follows: multi-method: 1, 2, 5, 10, 20 and 50 ng mL^{-1}; glyphosate/AMPA: 0.005, 0.01, 0.02, 0.05, 0.1, 0.2, 0.5, 1 and 2 μg mL^{-1} and GC-MS/MS: 0.5, 2.5, 5, 10, 25, 50 and 100 ng mL^{-1} respectively) The calibration curves presented satisfactory linearity of response versus concentration, with correlation coefficients above 0.99 and residuals of response lower than ±20%

In order to obtain high selectivity and sensitivity with respect to LC/MS analyses, selected reaction monitoring (SRM) was chosen as the data acquisition mode. Two identification ions (one precursor ion and one product ion) were used for each analyte. The relative response between two SRM transitions together with their retention times and peak shape of the respective reference standard (or of the isotopically labelled internal standard, in the case of glyphosate and AMPA) were used as a criterion for the identification of the compounds. Agilent Mass Hunter quantitation software was used for these purposes. The uncertainty of the ratio of the quantification ion to the qualifier ion of individual analytes was set at 20% of the expected values according to the standard evaluation procedure of Mass Hunter software. The quantitation of the analytes was performed using a matrix matched calibration standard or the internal standard calibration method in the case glyphosate and AMPA. Internal standards (deuterated glyphosate and AMPA) were added to all samples for these purposes.

The instrumental limits of quantification were determined from the injected amounts of standard solutions that provided a signal to noise ratio of 10. The limits of detection were determined from the amounts of analytes that provided a signal to noise ratio of 3. The repeated analysis ($n = 6$) of standard solutions was performed for these purposes. A LOQ of 0.004 mg kg^{-1} was achieved for the pesticide residues measured by the LC-MS/MS-based multi-method while for the compounds measured by GC-HRMS this LOQ was of 0.001 mg kg^{-1}, and for glyphosate and AMPA this was 0.05 mg kg^{-1}. The list of compounds analysed by LC-MS/MS-based multi-method and by GC-HRMS is presented in Table S13 and Table S14, respectively.

A recovery test was conducted as a part of accuracy validation by spiking soil samples (i.e., agricultural soils from a previous study that did not contain any of the tested residues) having concentration of pesticides near the LOQ, using 5 determinations at 2 concentration levels over the range of the calibration curve. For the LC-MS/MS-based multi-method the spiking levels were 0.004 and 0.04 mg kg^{-1}, for the GC-HRMS-based multi-method, 0.001 and 0.04 mg kg^{-1}, and for glyphosate and AMPA determinations, 0.05 and 0.50 mg kg^{-1}. The recoveries obtained in the fortified soils were between 70 and 120%. The validation parameters are listed in Table S13 and Table S14.

5. Histogram of the number of detected PPP residues per sample

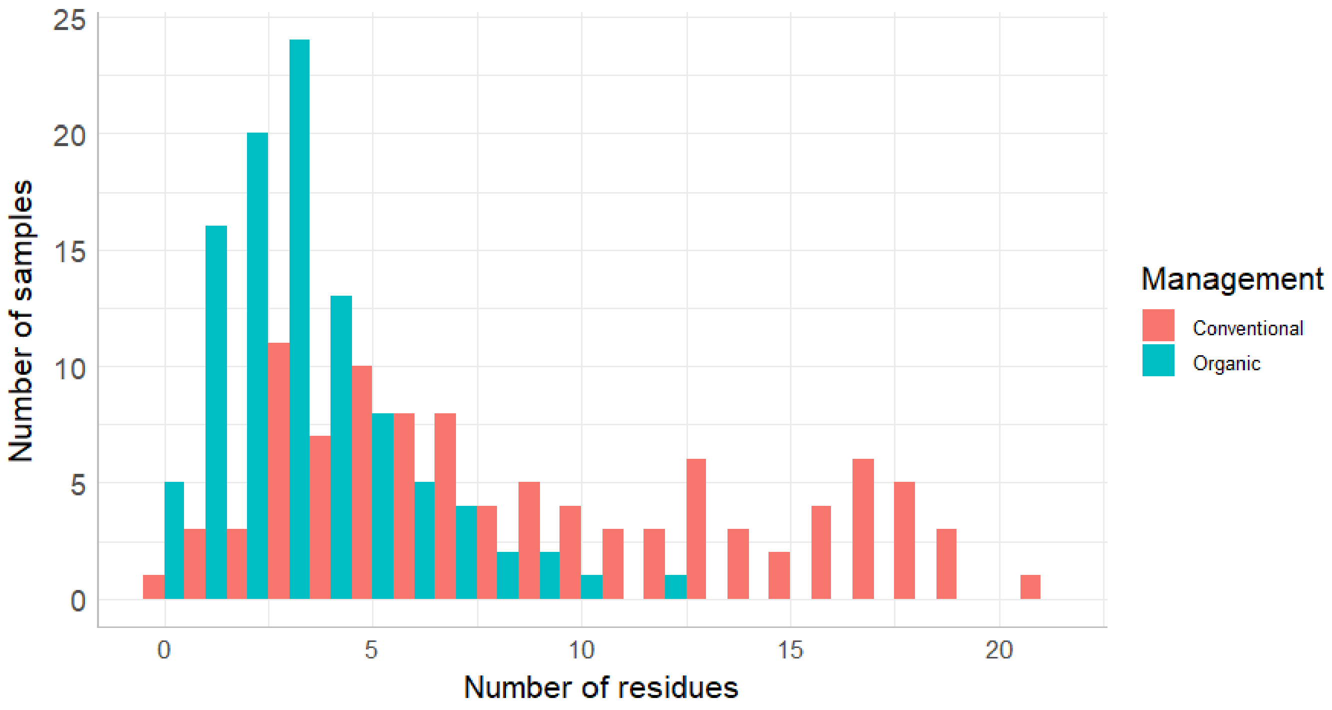

Figure S1 Histogram of the number of detected PPP residues per sample by farming type.

6. Figure S2 total concentrations of PPP residues by crop and region

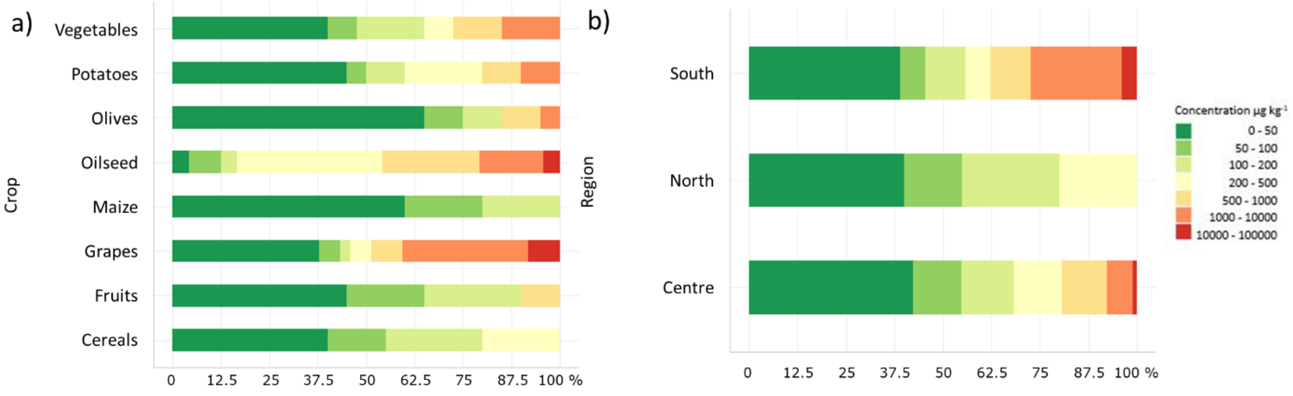

Figure S2a) Total PPP residue concentrations of the different crops. b) Pesticide residue concentrations of different climatic regions), South (France, Portugal, Italy and Spain), North (Denmark) and Centre (the Netherlands, Czech Republic, Switzerland, Slovenia and Croatia).

References:

(1) International Organization for Standardization [ISO]. EN ISO 11272:2017 Soil Quality - Determination of Dry Bulk Density. 2017.

(2) Ballabio, C.; Panagos, P.; Monatanarella, L. Mapping Topsoil Physical Properties at European Scale Using the LUCAS Database. *Geoderma* **2016**, *261*, 110–123. https://doi.org/10.1016/j.geoderma.2015.07.006.

(3) van Reeuwijk, L. P. Procedures for Soil Analysis. International Soil Reference and Information Centre (ISRIC) Technical Paper, No. 9: Wageningen 2002, p 19.

(4) Yang, X.; Wang, F.; Bento, C. P. M.; Xue, S.; Gai, L.; van Dam, R.; Mol, H.; Ritsema, C. J.; Geissen, V. Short-Term Transport of Glyphosate with Erosion in Chinese Loess Soil - A Flume Experiment. *Science of the Total Environment* **2015**, *512–513*, 406–414. https://doi.org/10.1016/j.scitotenv.2015.01.071.

(5) Bento, C. P. M.; Yang, X.; Gort, G.; Xue, S.; van Dam, R.; Zomer, P.; Mol, H. G. J.; Ritsema, C. J.; Geissen, V. Persistence of Glyphosate and Aminomethylphosphonic Acid in Loess Soil under Different Combinations of Temperature, Soil Moisture and Light/Darkness. *Science of the Total Environment* **2016**, *572*, 301–311. https://doi.org/10.1016/j.scitotenv.2016.07.215.

(6) Pihlström, T.; Fernández-Alba, A. R.; Gamón, M.; Amate, C. F.; De Kok, A.; O´regan, F.; Pelosi, P.; Mol, H.; Jezussek, M. *ANALYTICAL QUALITY CONTROL AND METHOD VALIDATION PROCEDURES FOR PESTICIDE RESIDUES ANALYSIS IN FOOD AND FEED*.
